# Hybrid Control of Digital Baker Map with Application to Pseudo-Random Number Generator

**DOI:** 10.3390/e23050578

**Published:** 2021-05-08

**Authors:** Yuhui Shi, Yashuang Deng

**Affiliations:** The School of Information and Safety Engineering, Zhongnan University of Economic and Law, Wuhan 430073, China; 202011200015@stu.zuel.edu.cn

**Keywords:** chaos, digital Baker map, dynamical degradation, hybrid control, entropy

## Abstract

Dynamical degradation occurs when chaotic systems are implemented on digital devices, which seriously threatens the security of chaos-based cryptosystems. The existing solutions mainly focus on the compensation of dynamical properties rather than on the elimination of the inherent biases of chaotic systems. In this paper, a unidirectional hybrid control method is proposed to improve the dynamical properties and to eliminate the biases of digital chaotic maps. A continuous chaotic system is introduced to provide external feedback control of the given digital chaotic map. Three different control modes are investigated, and the influence of control parameter on the properties of the controlled system is discussed. The experimental results show that the proposed method can not only improve the dynamical degradation of the digital chaotic map but also make the controlled digital system produce outputs with desirable performances. Finally, a pseudorandom number generator (PRNG) is proposed. Statistical analysis shows that the PRNG has good randomness and almost ideal entropy values.

## 1. Introduction

Chaos was first discovered by accident in 1961 while Lorenz was researching weather forecasting simulations [1]. However, the term “chaos” was only officially defined in 1975. Chaos is widely used in communication, mathematics, biology, information technology, economics, etc. primarily due to its distinct characteristics, such as sensitivity to an initial value, topological transitivity, ergodicity, unpredictability, etc. Specifically, the high consistency between chaos and cryptographic design criteria allowed for large numbers of chaos-based secure applications to emerge. Since the first chaotic stream cipher scheme was proposed in 1989 [2], chaotic encryption technology has been of great concern. Meanwhile, the design and analysis of chaotic cipher have been carried out one after another. Thus far, many secure communication systems have been proposed, including multimedia digital encryption [3], data watermarking, synchronization security systems, and data hiding systems based on chaos, etc.

Existing chaotic cryptography schemes are mostly based on chaotic systems implemented on digital devices with limited precision, namely digital chaotic systems. However, chaos collapses in the digital world, specifically, chaotic systems display dynamical degradation behaviors, such as short periodic orbits, low complexity, strong correlation, uneven distribution, etc. The occurrence of these situations make the output of the chaotic system reflect these system characteristics. Due to these system characteristics, the attacker can effectively capture the system orbit information to analyze the chaotic system; therefore, the chaotic system can be attacked and the chaotic cryptographic system poses a great safety hazard. Therefore, estimating these biases of the chaotic system is the core of ensuring the security of the chaotic cryptographic system. The “bias” is mainly observed from a security point of view, which refers to these non-random features of the original chaotic system such as the uneven distribution of outputs, the obvious attractor structure, among others. Such bias of a chaotic system often allows its outputs to reflect the system characteristics (such as information about the system parameters). In conclusion, an anti-degradation chaotic system plays an important role in the fields of cryptography and secure communication and it is of great significance to guarantee the security of chaotic security systems.

In view of the finite precision effect of digital chaotic systems, some solutions have been proposed to combat the degradation of digital chaotic systems. The first is to use high finite precision [4]. Wheeler and Matthews [5] proposed that the use of supercomputers and other hardware devices with higher computational accuracies would increase the period of the digital chaotic sequence but could not solve the essential problem. The second is to cascade multiple chaotic systems [6,7,8,9]. Multiple identical or different chaotic systems are cascaded to extend the period of a digital chaotic sequence and to resist predictability [10]. It is more unpredictability and more complex compared with the original chaotic system and has a larger parameter space. However, this method cannot entirely solve the problem of a short orbital period and the distribution of the output is not uniform. The third solution is to switch between multiple chaotic systems, which can indeed increase the average period duration but performs poorly in the improvement of the distribution [11]. More importantly, the final effect of this scheme depends on the switching rule and the switching system to a great extent, such that it is difficult to guarantee universality. The fourth is the perturbation mechanism [12,13,14,15,16,17,18,19,20,21,22]. Liu and Lin [23] created perturbation chaotic systems to perturb state variables and system parameters with a logistic map. Liu and Luo [24] proposed a continuous Chen system to perturb both the parameters and the inputs of the Chebyshev system. The results showed that the chaotic performance of the perturbed system is indeed enhanced, but there are still some problems such as unbalanced distribution. The fifth solution is a coupling mechanism [25,26,27,28]. Coupling can be single coupling or bidirectional coupling (two chaotic systems coupled to one another). The chaotic system can be analog or digital. Additionally, an external nonlinear source can be coupled to the chaotic system, such as a pseudorandom sequence or linear feedback shift register (LFSR) sequence or nonlinear feedback shift register (NFSR). Chen [29] proposed a state feedback control method that can be applied not only to one-dimensional but also multidimensional chaotic mapping. Deng [30] put forward an effect chaotification method for digital chaotic systems based on the differential mean value theorem and state feedback technology that can produce the desirable dynamical behaviors in terms of cryptography; however, it has a limitation with regard to the dimensions of chaotic systems. Zhang [31] proposed using piecewise linear chaotic mapping and cubic S-box coupling. Liu [32] proposed that the state variables of one digital chaotic map can be used to control the parameters of another digital chaotic map. In general, the coupling method has a good effect on the improvement of system performance.

It is clear from existing research that improvements in the performance of controlled systems depend on the control source and control mode. Indeed, continuous chaotic systems can preserve their good dynamical properties; however it is difficult to maintain long-term, stable synchronization owing to parameter uncertainties. Most chaotic cryptographic algorithms are designed based on digital chaotic systems mainly due to its reproducibility and stability. Unfortunately, although digital chaotic systems can easily retain synchronization for a long time given the same system and initial value, they often suffer from dynamical degradation due to finite computing precision. Additionally, such degeneration still exists for many autonomous digital chaotic systems, which means that an external control is needed to obtain a digital chaotic system with desirabled performance. From the above consideration, we discuss the unidirectional hybrid control model by introducing a continuous chaotic system. This paper introduces a continuous system to exert feedback control for the given digital chaotic map, where a Chen system [33] and a Baker system are chosen here. As a two-dimensional system, the Baker system is widely used in image encryption. Therefore, it is necessary to improve the digital system. We present the related literature on Baker maps [34]. The authors of [35] controlled Baker mapping based on the probabilistic coupling of controlled dynamics and control systems, and the subsequent improvement of coupled dynamics in a suitable functional space. The lifted dynamics is governed by linear Perron-Frobenius and Koopman operators. The novelty of [36] is that they described linear systems as microchaos. They showed that these vibrations may be related to the deterministic chaotic dynamics caused by sampling and quantization. In addition, they gave a detailed chaotic analysis as proof for the PD controlled oscillator. In this case, they developed a method to accurately calculate the average lifetimes of chaotic transients. Three control modes were investigated, and the effect of the control parameter on system performance was also discussed. Their experimental simulations showed that not only was the skewness in dynamic performance of the controlled system eliminated but also the desired dynamic performance was presented. In particular, it is worth mentioning that the outputs displayed almost ideal information entropy, approximate entropy, and permutation entropy. Finally, a novel PRNG was constructed based on the controlled digital Baker map. Statistical tests along with other analyzes were carried out to show the randomness of the generated sequences.

This paper is organized as follows. Section 2 briefly introduces the degradation phenomenon of the digital Baker map. Section 3 discusses three control modes and analyzes the effect of control gain coefficient on the output control mode. Section 4 gives a performance comparison with existing methods. Section 5 presents a novel PRNG and analyzes its randomness and security.

## 2. Dynamical Degradation of Digital Baker Map

The Baker chaotic map can be written as follows:

(1) $$\left(x_{i+1},y_{i+1}\right)=\left\{\begin{array}{cc} (\frac{x_{i}}{u},uy_{i}) & 0<x_{i},y_{i}\leq u\\ (\frac{x_{i}-u}{1-u},\left(1-u\right)y_{i}+u) & u<x_{i},y_{i}\leq 1 \end{array}\right.$$

where $x_{i},y_{i}\in \left[0,1\right]$ are state variables of the system and u∈(0,1] is the system parameter.

When the Baker chaotic map is realized with finite computing precisions, it degrades into the following digital map:

(2) $$\left(x_{{}_{i+1}}^{*},y_{{}_{i+1}}^{*}\right)=\left\{\begin{array}{cc} \left(\frac{x_{{}_{i}}^{*}}{u},uy_{{}_{i}}^{*}\right) & 0<x_{{}_{i}}^{*},y_{{}_{i}}^{*}\leq u\\ \left(\frac{x_{{}_{i}}^{*}-u}{1-u},\left(1-u\right)y_{{}_{i}}^{*}+u\right) & u<x_{{}_{i}}^{*},y_{{}_{i}}^{*}\leq 1 \end{array}\right.$$

where $x_{i+1}^{*}=f\left(x_{i}^{*}\right)$ and $x_{i}^{*}=FL\left(x_{i}\right)$. $x_{i}^{*}\in \Omega_{p}$ is a digital variable with p bit precision, and $x_{i}\in \Omega$ is a real-intermediate variable. $\Omega_{p}=\left\{x_{i}=k\times 2^{-p}|k=0,1,2,...,2^{p-1}\right\}$. *p* is the computing precision. f:Ω→Ω is a nonlinear function. $FL:\Omega \to \Omega_{p}$ is a quantization function, and it is defined as $FL\left(x\right)=\left\lfloor x\cdot 2^{p}\right\rfloor \cdot 2^{-p}$. The quantification method of variable *y* is the same as that of variable *x*.

We set *p* = 8. Indeed, the precision can, of course, be selected several times larger in practical applications. There are two reasons why we chose a precision of 8. First, the lower the precision, the more obvious the degeneration behavior of the chaotic system. Our solution can still maintain good chaotic performance even under low-precision situations, which will undoubtedly show the effectiveness of our method. Second, the higher the precision, the longer it takes to implement with digital equipment. Therefore, it is necessary to study the realization of digital chaotic system with low precision. We choose parameter and initial values *u* = 0.49, $x_{0}$ = 0.2, and $y_{0}$ = 0.29 randomly. These parameters and initial values were only randomly selected by us, and the effect of our scheme is the same for other values. We observed the dynamical properties of the digital Baker map.

**Figure 1:**
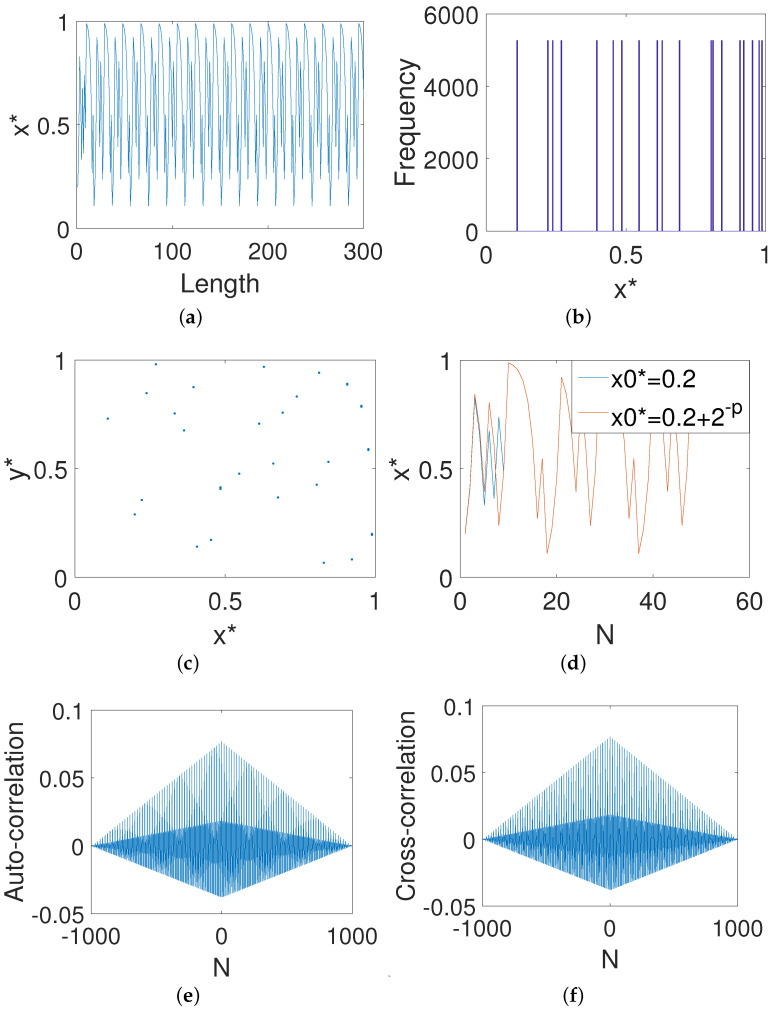
The dynamical properties of the digital Baker map. (**a**) The *x*-dimensional output. (**b**) The *x*-dimensional distribution. (**c**) Phase diagram. (**d**) Initial value sensitivity. (**e**) Auto-correlation. (**f**) Cross-correlation.

It is clear from Figure 1a that the digital map enters a periodic loop state only after nearly 40 iterations. As shown in Figure 1b,c, the digital map has obvious distribution characteristics and architectural features. Such dynamical biases often make the system vulnerable to attacks. Meanwhile, it can be seen from Figure 1d that two orbits meet only after 10 iterations, which indicates that the system has no sensitivity to initial values. Moreover, both the bad auto-correlation shown in Figure 1e and the cross-correlation shown in Figure 1f indicate that strong correlations between different outputs of the system exist.

Moreover, three typical types of entropy indicators are applied to investigate the complexity of the digital Baker system from different aspects. Information entropy measures the uncertainty or unpredictability of a time series. Approximate entropy primarily measures the probability of generating a new pattern in a time series. Permutation entropy depicts the structural complexity of a time series: the closer the permutation entropy is to 1, the better the randomness of the sequence. In general, the smaller the entropy is, the simpler and more regular the time series. On the contrary, the larger the entropy value, the more complex and random the time series.

**Figure 2:**
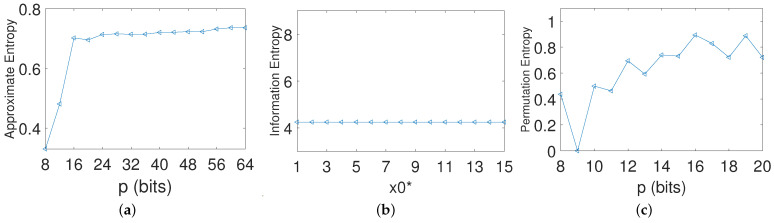
Entropy analysis of the digital Baker map. (**a**) Approximate entropy. (**b**) Information entropy. (**c**) Permutation entropy.

It can be seen from Figure 2a that the approximate entropy of the digital Baker system at different precisions (except 8–12 bits) is about 0.7. This shows that the complexity of the system is low and that the regularity is strong. Under the precison of 8, the closer the information entropy is to 8, the better the randomness of the sequence. Figure 2b shows that the information entropy of the digital Baker system is about 4.2, which means that it has poor randomness. Figure 2c shows that the permutation entropy of the digital Baker mapping at different precisions is almost 0.4 to 0.8.

From the above analysis, it can be included that the original good characteristics disappear indeed and that some dynamical degradation behaviors occur when the Baker chaotic map is implemented with finite computing precisions, including the periodic orbit, low complexity and strong correlation, uneven distribution, etc.

## 3. A Unidirectional Hybrid Control Scheme

In this section, a unidirectional hybrid control scheme is set up, where a continuous Chen chaotic system is introduced to control external state feedback for the digital Baker map.

### 3.1. Chen Chaotic System

A continuous Chen system can be defined as follows:

(3) $$\left\{\begin{array}{c} \dot{x}=a\left(y-x\right)\\ \dot{y}=\left(c-a\right)x-xz+cy\\ \dot{z}=xy-bz \end{array}\right.$$

**Figure 3:**
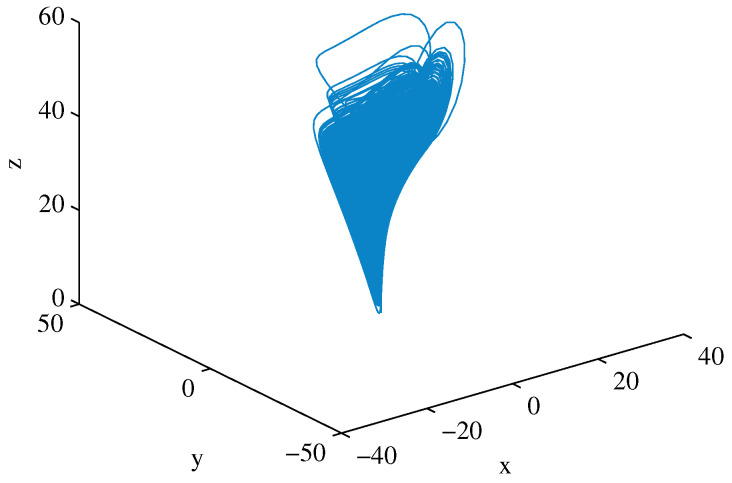
Chen chaotic attractor.

It is well known that the system has a chaotic attractor when a=35, $b=\frac{8}{3}$ and c=28, as shown in Figure 3.

### 3.2. Three Control Modes

In this part, we first investigate the performances of three control modes, including parameter control, input control, and output control of the digital Baker map. Then, the optimal control mode is chosen to construct the final control model.

The first control mode controls the parameter *u* of the digital map, which can be represented as follows:

(4) $$\begin{array}{cc} u\to u_{i}:u_{i+1}=u_{i}+{\tilde{z}}_{i} & \;\mathrm{mod}\;1 \end{array}$$

where ${\tilde{z}}_{i}$ denotes the sampling state of a Chen chaotic system in *z* dimension.

Then, the parameter-controlled digital Baker map (PCB) is expressed as Equation (5):

(5) $$\left(x_{i+1},y_{i+1}\right)=\left\{\begin{array}{cc} \left(\frac{x_{{}_{i}}^{*}}{u_{i}},y_{{}_{i}}^{*}\cdot u_{i}\right) & 0<x_{i}^{*},y_{i}^{*}\leq u_{i}\\ \left(\frac{x_{{}_{i}}^{*}-u_{i}}{1-u_{i}},y_{{}_{i}}^{*}\cdot \left(1-u_{i}\right)+u_{i}\right) & u_{i}<x_{i}^{*},y_{i}^{*}\leq 1\\ x_{i}^{*}=FL\left(x_{i}\right) & \\ y_{i}^{*}=FL\left(y_{i}\right) & \end{array}\right.$$

The second control mode controls the inputs *x* and *y* of the digital map, which can be represented as follows:

(6) $$\begin{array}{cc} {\overset{-}{x}}_{i}=x_{{}_{i}}^{*}+{\tilde{x}}_{i} & \;\mathrm{mod}\;1 \end{array}$$

(7) $$\begin{array}{cc} {\overset{-}{y}}_{i}=y_{{}_{i}}^{*}+{\tilde{y}}_{i} & \;\mathrm{mod}\;1 \end{array}$$

where ${\tilde{x}}_{i}$ and ${\tilde{y}}_{i}$ denote the sampling states of a Chen chaotic system in the *x* and *y* dimensions.

Then, the input-controlled digital Baker map (ICB) is expressed as follows:

(8) $$\left(x_{i+1},y_{i+1}\right)=\left\{\begin{array}{cc} \left(\frac{{\bar{x}}_{i}}{u},{\bar{y}}_{i}\cdot u\right) & 0<\bar{x_{i}},{\bar{y}}_{i}\leq u\\ (\frac{{\bar{x}}_{i}-u}{1-u},{\bar{y}}_{i}\cdot \left(1-u\right)+u & u<\bar{x_{i}},{\bar{y}}_{i}\leq 1\\ {\bar{x}}_{i}=FL\left(x_{i}\right) & \\ {\bar{y}}_{i}=FL\left(y_{i}\right) & \end{array}\right.$$

The third control mode controls the outputs of the digital map, and the output-controlled Baker chaotic system (OCB) is expressed as follows:

(9) $$\left(x_{i+1},y_{i+1}\right)=\left\{\begin{array}{ccc} \left(\frac{x_{{}_{i}}^{*}}{u}+{\tilde{x}}_{i},y_{{}_{i}}^{*}\cdot u+{\tilde{y}}_{i}\right) & \;\mathrm{mod}\;1 & 0<x_{{}_{i}}^{*},y_{{}_{i}}^{*}\leq u\\ \left(\frac{x_{{}_{i}}^{*}-u}{1-u}+{\tilde{x}}_{i},y_{{}_{i}}^{*}\cdot \left(1-u\right)+u+{\tilde{y}}_{i}\right) & \;\mathrm{mod}\;1 & u<x_{{}_{i}}^{*},y_{{}_{i}}^{*}\leq 1\\ x_{i}^{*}=FL\left(x_{i}\right) & & \\ y_{i}^{*}=FL\left(y_{i}\right) & & \end{array}\right.$$

### 3.3. Performance Comparison of Three Control Modes

Set the precision *p* = 8, system parameter *u* = 0.49, and initial values $x_{0}$ = 0.81 and $y_{0}$ = 0.29. Then, we compared three control modes by analyzing the dynamical properties of three controlled digital Baker maps including the orbit, distribution, phase diagram, sensitivity to initial states, correlation, and entropy of the control systems.

#### 3.3.1. Trajectory

**Figure 4:**
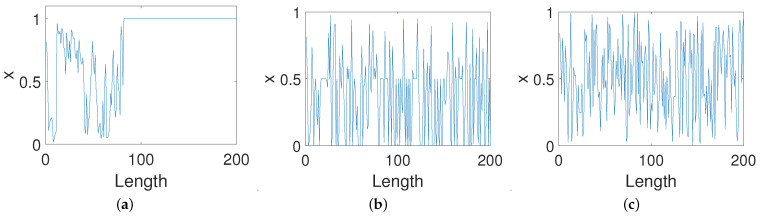
Trajectories of systems under three control modes. (**a**) PCB. (**b**) ICB. (**c**) OCB.

As shown in Figure 4a, the trajectory of Equation (5) quickly converges to a fixed point after almost 90 iterations, which is slightly better than the original digital map. Figure 4b shows that the trajectory of Equation (8) converges to a cycle or a fixed point after a few iterations. By contrast, it is obvious from Figure 4c that it is difficult to find a period in the trajectory of Equation (9) that shows that the OCB mode can greatly extend the period of the digital system and that performs better than the other two control modes.

#### 3.3.2. Frequency Distribution

**Figure 5:**
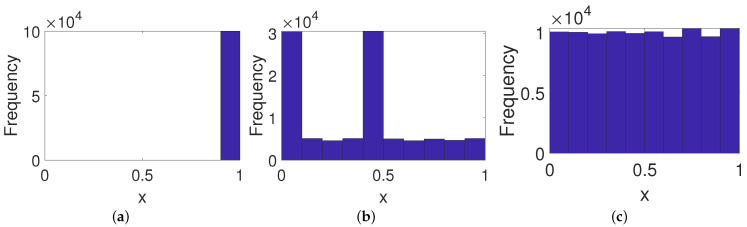
Frequency distributions of systems under three control modes. (**a**) PCB. (**b**) ICB. (**c**) OCB.

As shown in Figure 5a, the frequency distribution of the PCB system becomes worse than the original digital system since there is a fixed point. Meanwhile, it is clear from Figure 5b that there is an obvious characteristic in the frequency distribution of the ICB system. On the contrary, the frequency distribution of the OCB system reveals an almost even distribution (see Figure 5c), which indicates the OCB behaves better than the other two control modes in this case.

#### 3.3.3. Phase Diagram

**Figure 6:**
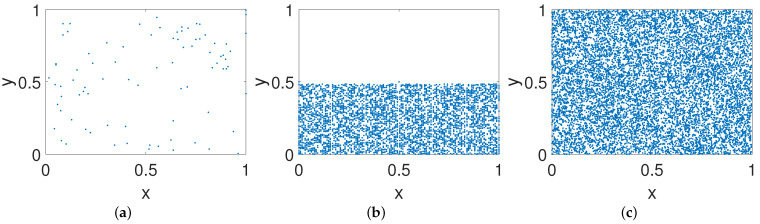
Phase diagrams of systems under three control modes. (**a**) PCB. (**b**) ICB. (**c**) OCB.

As shown in Figure 6a, the PCB mode does not seem to improve the phase space of digital system. It can be seen from Figure 6b that the states cannot go through the entire phase space. Meanwhile, no pattern exists and only a noise-like phenomenon in the phase diagram of OCB system exists (see Figure 6c), which prevents any relevant information from the phase diagram. In this way, the OCB mode behaves better than two other control modes.

#### 3.3.4. Auto-Correlation

**Figure 7:**
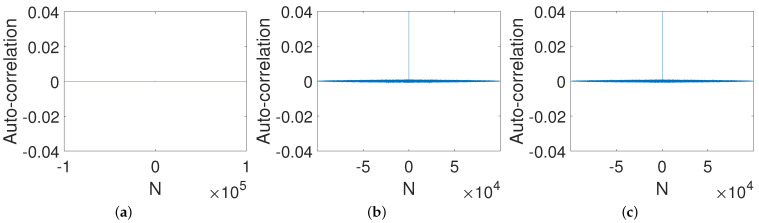
Auto-correlations of systems under three control modes. (**a**) PCB. (**b**) ICB. (**c**) OCB.

We investigated the auto-correlation of output generated from three controlled systems; the result is shown in Figure 7. Obviously, all three control modes performed well in improving the correlation between outputs of the systems due to the delta-like auto-correlation.

#### 3.3.5. Sensitivity to Initial Condition

**Figure 8:**
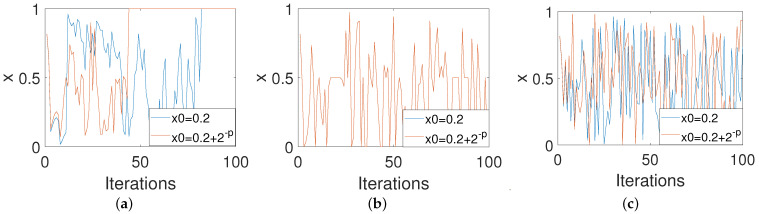
The sensitivity properties to the initial values of systems under three control modes. (**a**) PCB. (**b**) ICB. (**c**) OCB.

We are also concerned with the performances of the three control modes in improving the property of sensitivity to the initial conditions of digital systems. Consider two different orbits generated from two slightly different initial values of 0.84 and $0.84+2^{-8}$. It can be seen from Figure 8a that two orbits converge after 80 iterations for the PCB system. Figure 8b shows that two orbits overlap for the ICB system. Figure 8c shows that these are two completely different orbits for the OCB system. This means that the OCB system clearly conforms to the characteristics of a chaotic system.

According to the above analysis, it can be concluded that OCB performs better than the other two control modes in improving the dynamical properties of the digital Baker map. The authors of [17] also proved that the disturbance of the output is more effective than the disturbance of the input and the parameters. Therefore, we use the OCB mode to carry out our work in the following sections.

### 3.4. Model Optimization

In this section, we try to add a control gain coefficient *d* to the OCB mode to obtain the optimal control model. The new system is defined as Equation (10):

(10) $$\left(x_{i+1},y_{i+1}\right)=\left\{\begin{array}{ccc} \left(\frac{x_{{}_{i}}^{*}}{u}+d\cdot {\tilde{x}}_{i},y_{{}_{i}}^{*}\cdot u+d\cdot {\tilde{y}}_{i}\right) & \;\mathrm{mod}\;1 & 0<x_{{}_{i}}^{*},y_{{}_{i}}^{*}\leq u\\ \left(\frac{x_{{}_{i}}^{*}-u}{1-u}+d\cdot {\tilde{x}}_{i},y_{{}_{i}}^{*}\cdot \left(1-u\right)+u+d\cdot {\tilde{y}}_{i}\right) & \;\mathrm{mod}\;1 & u<x_{{}_{i}}^{*},y_{{}_{i}}^{*}\leq 1\\ x_{i}^{*}=FL\left(x_{i}\right) & & \\ y_{i}^{*}=FL\left(y_{i}\right) & & \end{array}\right.$$

Set *p* = 8, $x_{0}$ = 0.2, $y_{0}$ = 0.29, and *u* = 0.49. We then analyze the dynamical properties of the digital OCB system qualitatively and quantitatively using various of indicators, including the frequency distribution, auto-correlation, cross-correlation, approximate entropy, information entropy, and permutation entropy.

#### 3.4.1. Frequency Distribution

**Figure 9:**
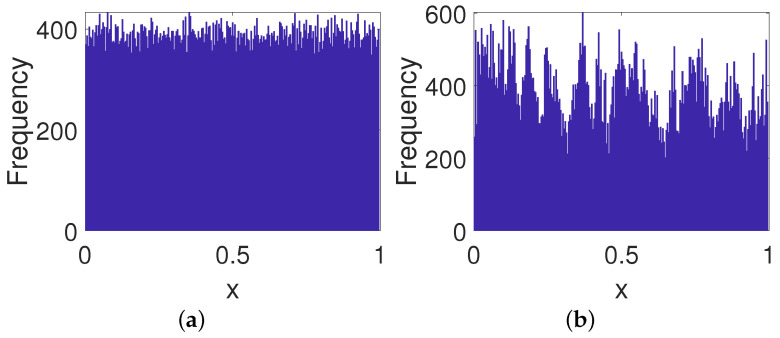

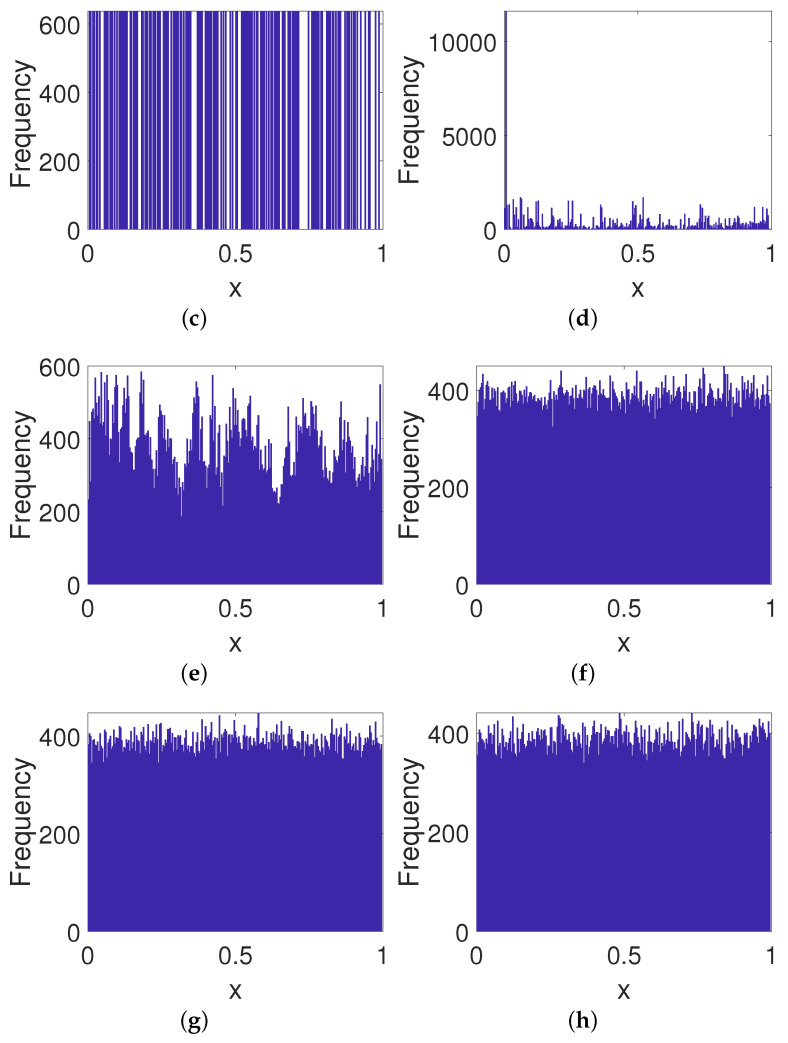
Frequency distributions of digital OCB systems with different gain coefficients. (**a**) *d* = −10. (**b**) *d* = −0.001. (**c**) *d* = 0. (**d**) *d* = 0.0001. (**e**) *d* = 0.001. (**f**) *d* = 0.01. (**g**) *d* = 1. (**h**) *d* = 10.

As shown in Figure 9, the distribution seems to depend only on the absolute value of the gain coefficient rather than its sign. Additionally, the distribution becomes almost uniform and essentially does not change anymore when the absolute value of gain coefficient is greater than 0.01.

#### 3.4.2. Auto-Correlation

**Figure 10:**
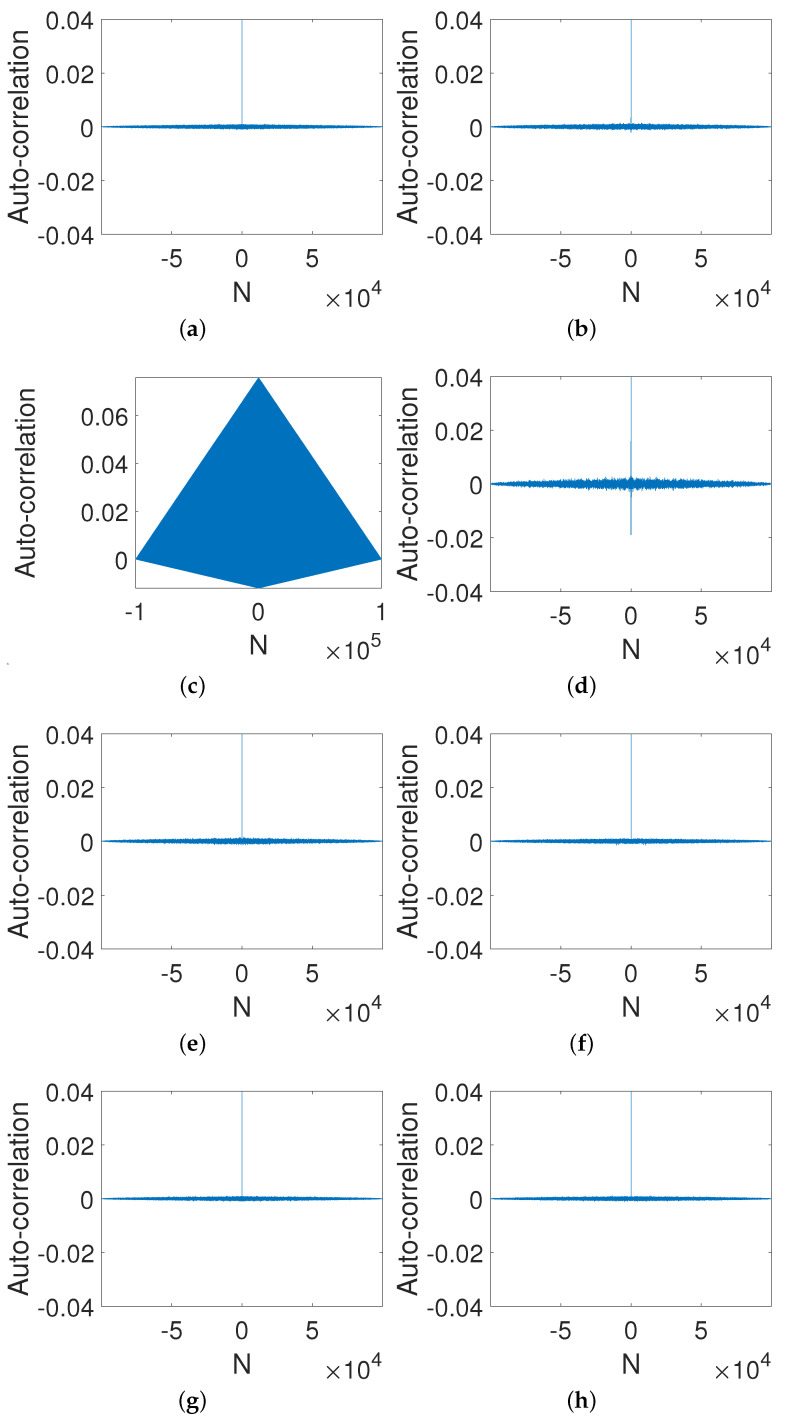
Auto-correlations of digital OCB systems with different gain coefficients. (**a**) *d* = −10. (**b**) *d* = −0.001. (**c**) *d* = 0. (**d**) *d* = 0.0001. (**e**) *d* = 0.001. (**f**) *d* = 0.01. (**g**) *d* = 1. (**h**) *d* = 10.

It is easy to see from Figure 10 that the auto-correlation is close to a delta-like function even through the gain coefficient is small, i.e., d=0.0001 (Figure 10d). In general, the larger the gain coefficient, the more uniform the distribution.

#### 3.4.3. Cross-Correlation

**Figure 11:**
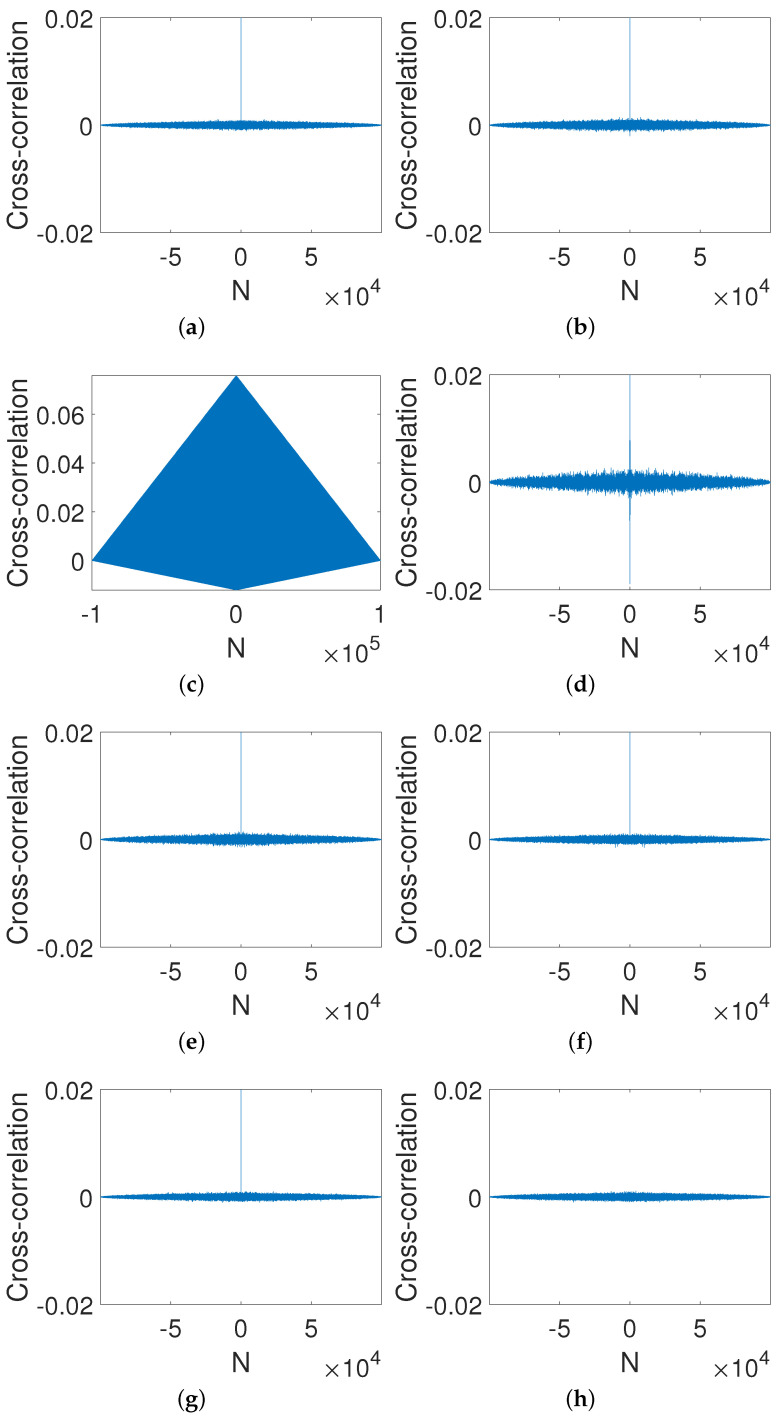
Cross-correlation of digital OCB systems with different gain coefficients. (**a**) *d* = −10. (**b**) *d* = −0.001. (**c**) *d* = 0. (**d**) *d* = 0.0001. (**e**) *d* = 0.001. (**f**) *d* = 0.01. (**g**) *d* = 1. (**h**) *d* = 10.

Figure 11 shows the correlation of two orbits with two slightly different initial values, i.e., $x{}_{0}=0.2$ and $x{}_{0}=0.2+2^{-p}$. It is clear that the cross-correlation of the two sequences can be improved as long as the gain coefficient is not zero. Additionally, the cross-correlation reaches almost zero when d=10, as we expected.

#### 3.4.4. Approximate Entropy

As shown in Table 1, there is a growing trend in the approximate entropy value with an increase in the gain coefficient *d*. Specifically, the approximate entropy is just as bad as that of the digital Baker system when *d* is small (such as for d=−0.001 and d=0.001) and exceeds two when the absolute value of *d* is larger than 10.

#### 3.4.5. Information Entropy

It can be seen from Table 2 that the information entropy was significantly improved (except when d=0.0001) compared with the digital system before control and almost reaches the maximum value 8 under the current computing precision. In this way, the complexity is improved greatly via hybrid output feedback control.

#### 3.4.6. Permutation Entropy

Permutation entropy depicts the structural complexity of a time series, and the value is usually between 0 and 1. It can be concluded from Table 3 that the greater the absolute value of the gain coefficient, the closer the entropy is to 1 and the more complex the sequence.

## 4. Dynamical Performance Comparison

### 4.1. Performance Comparison of the Systems before and after Control

Set p=8, $x_{0}=0.2$, $y_{0}=0.29$, u=0.49, and d=10. Then, we can observe the dynamical performance of the digital Baker map (2) and the final controlled system (10).

#### 4.1.1. Trajectories and Phase Diagrams

**Figure 12:**
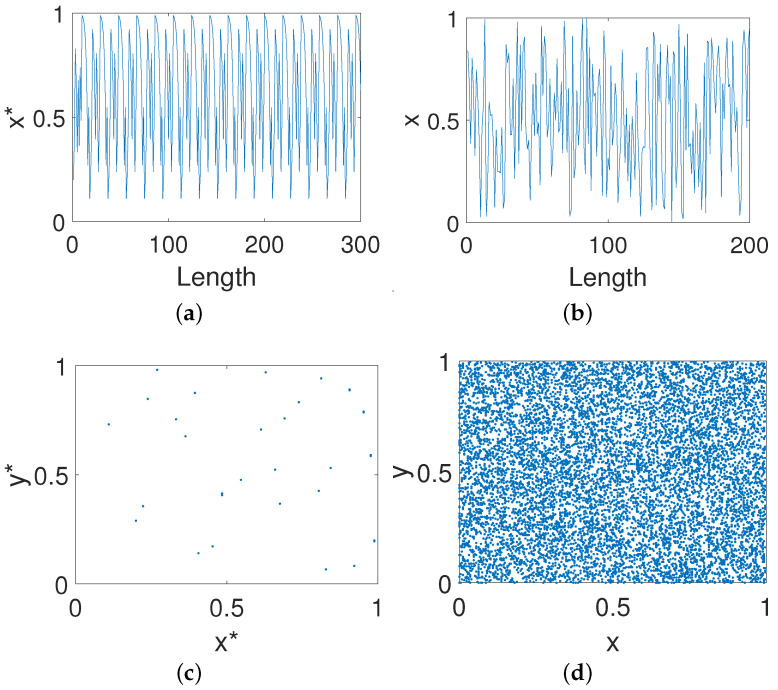
Trajectories and phase diagrams. (**a**) The x-dimensional trajectory of the digital Baker system. (**b**) The x-dimensional trajectory of the controlled system. (**c**) The phase diagram of the digital Baker system. (**d**) The phase diagram of the controlled system.

Figure 12 shows the trajectories and phase diagrams of the digital Baker map before and after output feedback control. Obvious short-period behaviors exist for the digital Baker map, but such a phenomenon can be eliminated after control. Meanwhile, the phase space of the digital OCB system is much more complex than that of the system before control. As shown in Figure 12d, there is no obvious structural patterns and seems completely "random" for the phase space of the system after control. In this way, we can say that the structural bias has been eliminated via the OCB mode with the selected control gain coefficient.

#### 4.1.2. Frequency Distribution

**Figure 13:**
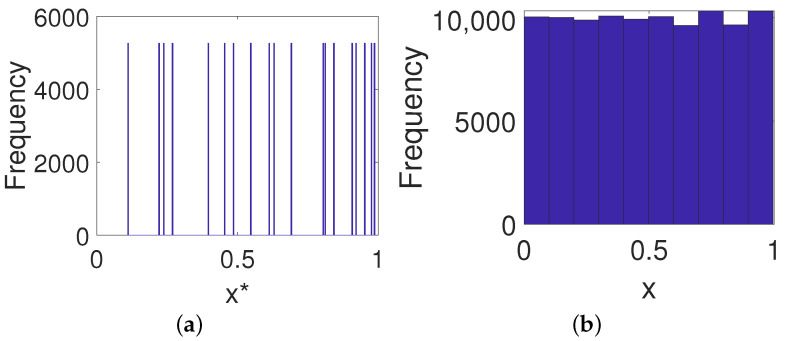
Frequency distribution. (**a**) Digital Baker system. (**b**) The controlled system.

It can be seen from Figure 13b that the digital Baker map displays almost uniform distribution even with lower precision after output feedback control, which undoubtedly enhances the resistance to statistical analysis.

#### 4.1.3. Correlation

**Figure 14:**
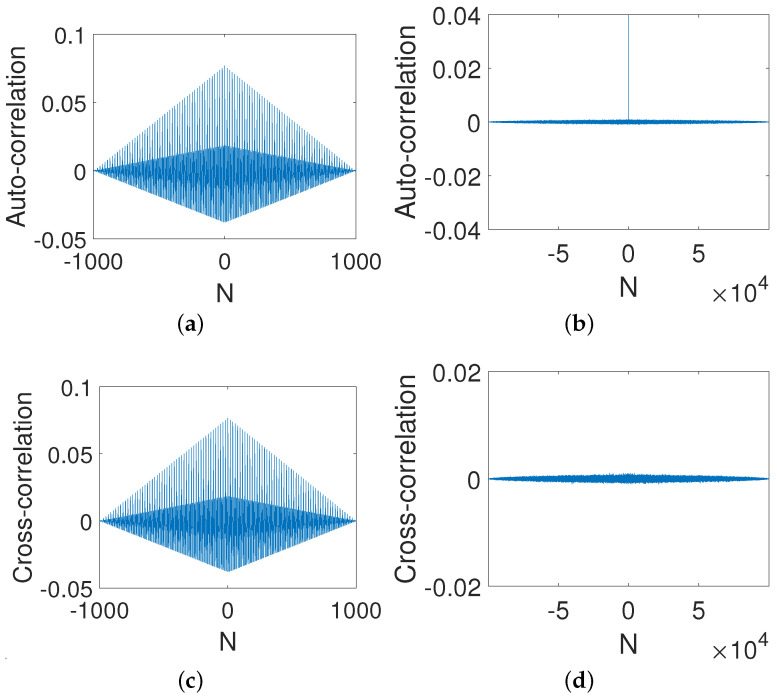
Auto-correlation and cross-correlation. (**a**) Auto-correlation of a digital Baker system. (**b**) Auto-correlation of a controlled system. (**c**) Cross-correlation of a digital Baker system. (**d**) Cross-correlation of a controlled system.

It is clear that the original strong correlation of the digital Baker map shown in Figure 14a was eliminated via output feedback control (see Figure 14b). More importantly, the controlled system displays desired correlation functions, i.e., a impulse-like auto-correlation function (see Figure 14c) and a close-to-zero cross-correlation function (see Figure 14d). This implies that the ability of the controlled system to resist a correlation attack can be enhanced greatly.

#### 4.1.4. Lyapunov Exponent

Lyapunov exponent refers to the average rate of exponential separation or convergence of two close orbits in phase space over time. It is one of the features used to identify several values of chaotic motion. A negative lyapunov exponent indicates convergence, while a positive lyapunov exponent demonstrates divergence and chaos. Divergence of the exponential law of the orbit means that the almost indistinguishable difference of the initial conditions is displayed quickly, thus quickly losing the possibility of the future state of the system being predicted. A lyapunov exponent greater than 0 means that the system is in a chaotic state. After testing, the maximum lyapunov exponen of our system in the x-direction is 1.518 and the maximum lyapunov exponent in the y-direction is 1.6952. This shows that our system is chaotic and has good initial sensitivity. However, the maximum lyapunov exponent of the digital Baker system is still negative, −0.0011 and −0.0035, respectively, which indicates that it is a periodic system.

#### 4.1.5. Entropy Analysis

We further observe the effect of computing precision and initial value on the complexity of the digital Baker map before and after control, respectively, by using approximate entropy, information entropy, and permutation entropy.

**Figure 15:**
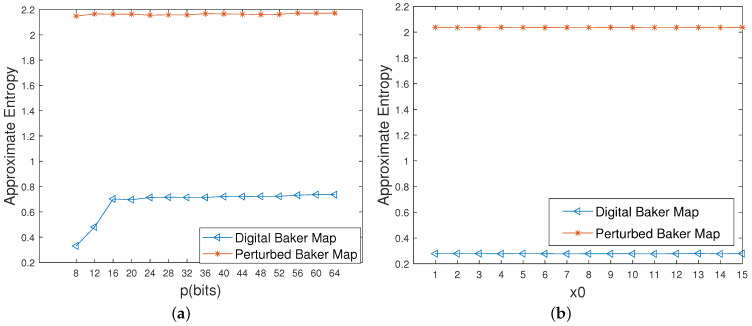
Approximate entropy of the digital Baker maps before and after control. (**a**) Different precisions. (**b**) Different initial values.

**Figure 16:**
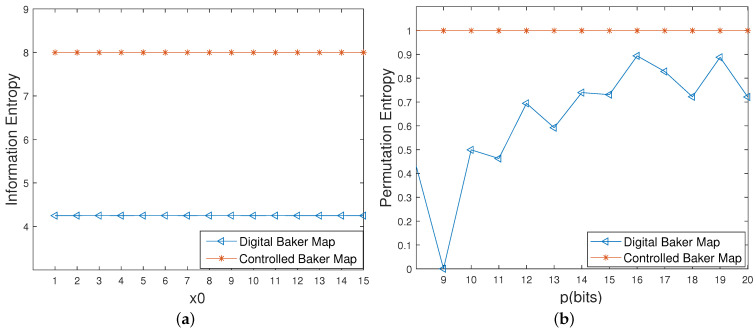
Information entropy and permutation entropy of the digital Baker maps before and after control. (**a**) Information entropies with different initial values. (**b**) Permutation entropies with different precisions.

It can be seen from Figure 15 that the approximate entropy becomes twice the original digital one. Meanwhile, all of the information entropy values of the controlled system reach the ideal value of 8 with different initial values, which are much higher than these of the original digital system, as depicted in Figure 16a. Moreover, as shown in Figure 16b, there is a growing trend in the permutation entropy of the original digital system with an increase in precision, but a big deviation from the ideal value of 1 still exists. However, the permutation entropy of the controlled system is close to 1 even with low computing precisions, which means that the complexity of the controlled system is much higher than that of the original digital system under any circumstances.

### 4.2. Comparison of the Proposed Control Scheme with Existing Methods

Some typical approaches to degradation of the digital chaotic system are introduced here to further show the efficiency of the proposed control method, including the novel double perturbation method (DPM) in [23], the dual perturb method (CCM) in [24], the delay-introducing method (DIM) in [26], the coupled chaotic model (CPM) in [32], the bit reversal method (BRM) in [15], the nested coupling models (2D-SCS) in [6], and the stochastic jumps model (SJM) in [13]. We set the precision to p=8, and we observed the performances of these methods.

#### 4.2.1. Trajectories

**Figure 17:**
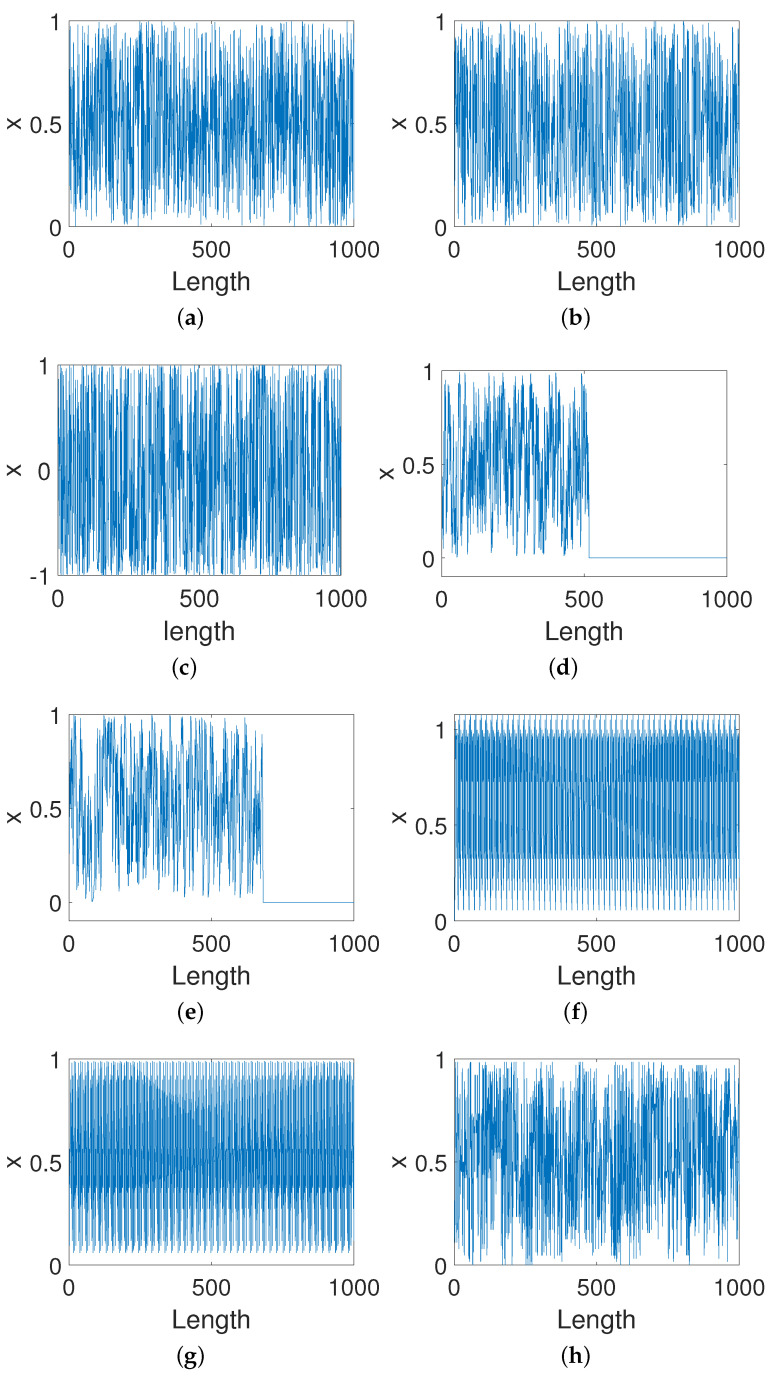
Trajectories of improved systems for different methods. (**a**) Our method. (**b**) DPM. (**c**) CCM. (**d**) DIM. (**e**) CPM. (**f**) BRM. (**g**) 2D-SCS. (**h**) SJM.

Figure 17 shows the trajectories of eight improved systems using existing methods and our scheme. It can be seen from Figure 17d,e that two improved systems converge to a fixed point 0 after a few hundred iterations via DIM and CPM. Additionally, there are obvious cycle phenomena in two improved systems, BRM and 2D-SCS, as shown in Figure 17f,g. Moreover, there is no discernible difference for other methods in extending the orbit of the system with the precision of 8 bits.

#### 4.2.2. Frequency Distribution

**Figure 18:**
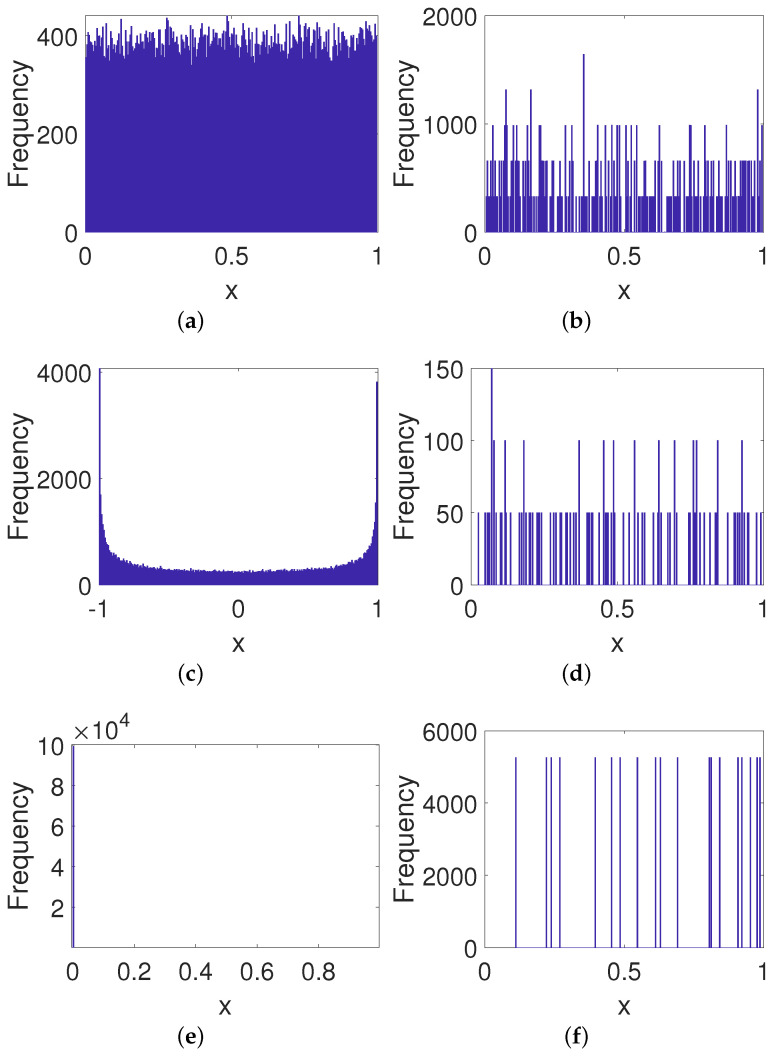

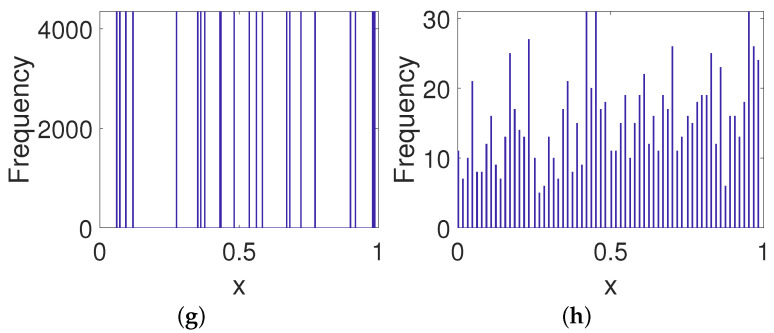
Frequency distributions of improved systems for different methods. (**a**) Our method. (**b**) DPM. (**c**) CCM. (**d**) DIM. (**e**) CPM. (**f**) BRM. (**g**) 2D-SCS. (**h**) SJM.

It can be observed from Figure 18 that the controlled system obtained using our method has an almost uniform distribution even with lower precision, which greatly improves the ability to resist attacks. By contrast, the improved system for the method of CCM shows a *U*-shaped distribution, i.e., an almost uniform distribution except for at both ends of the domain. Moreover, the improved systems for other methods show relatively poorer distributions. These significant distribution biases often make the system vulnerable to statistical attacks. Hence, our method has a better performance than others when enhancing the security of digital systems.

#### 4.2.3. Phase Diagram

**Figure 19:**
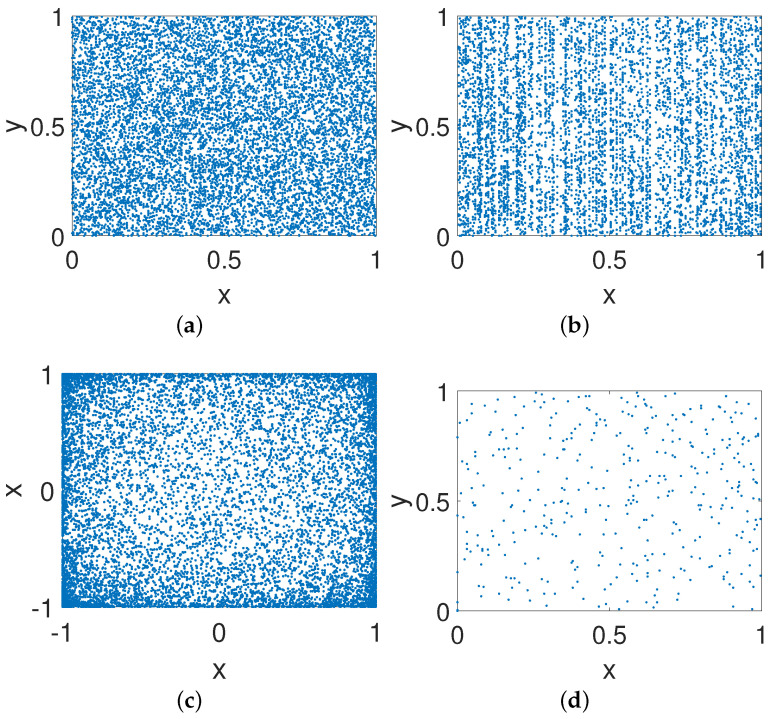

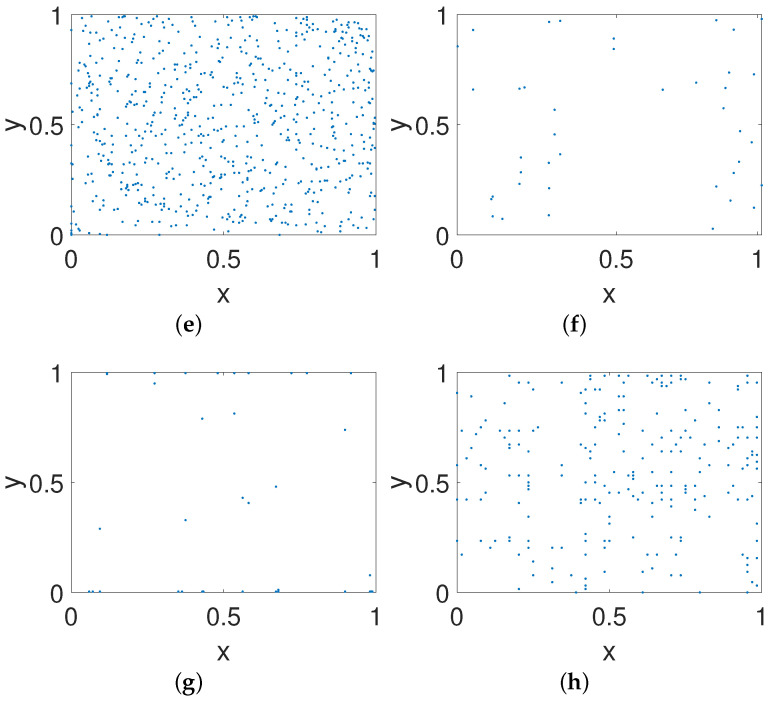
Phase diagrams of improved systems for different methods. (**a**) Our method. (**b**) DPM. (**c**) CCM. (**d**) DIM. (**e**) CPM. (**f**) BRM. (**g**) 2D-SCS. (h) SJM.

As shown in Figure 19a, the phase diagram of the controlled system for our method is a noise-like diagram, which can improve the security of the system indirectly. Meanwhile, the distributions shown in Figure 19b,c are relatively dense, but both still present specific distribution characteristics. Figure 19d–h are relatively scattered with low ergodicity. Hence, our method has better performance than others when eliminating the structural features of the given systems.

#### 4.2.4. Entropy Analysis

Given that the increase in the complexity of the system can be used as another effective indicator to evaluate different solutions to degradation, three entropy indicators are applied here to compare the efficiency of different methods.

**Figure 20:**
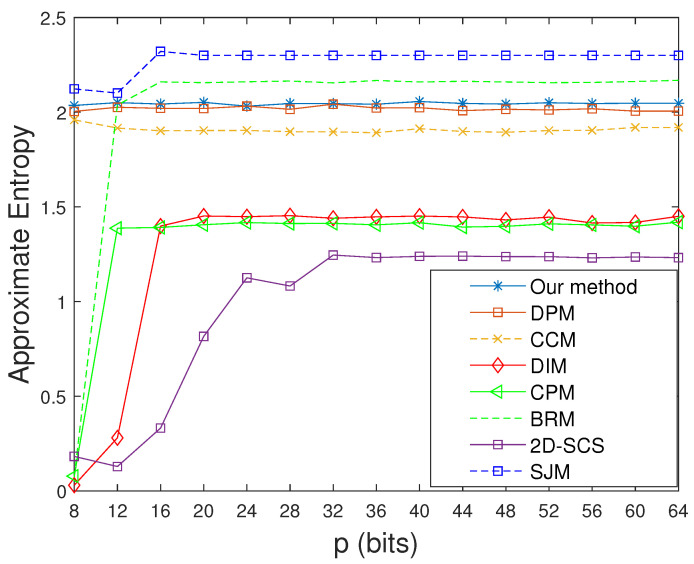
Approximate entropy values of improved systems for different methods.

As shown in Table 4, the information entropy of the controlled system via our method is extremely close to the ideal value of 8 under the precision of 8, which means that our system has greater uncertainty than other systems. It is clear from Figure 20 that the four improved systems using SJM, our method, DPM, and CCM not only have higher approximate entropy values but also entropy values that cannot change much with increasing in computing precision. This means that we can obtain stable entropy values even with lower precisions, which can reduce the implementation cost to some extent. By contrast, the improved systems for the other four methods—BRM, CPM, DIM, and 2D-SCS—display relatively lower and unstable approximate entropy values. Moreover, the permutation entropy of our controlled system is larger than others and extremely close to 1 (see Table 5), which means that the system has higher structural complexity.

From the above analysis, the conditions we set are relatively harsh, and our scheme can still maintain relatively good chaotic characteristics in this case, which highlights the advantages of our solution. However, it should be mentioned that the implementation complexity may be a little less than that for other methods due to the involvement of a continuous chaotic system. We discuss the balance between performance and implementation costs below.

## 5. Proposed Pseudorandom Bit Generator

In this section, we propose a new pseudorandom bit generator (PRBG) based on the above digital OCB system (Equation (10)). The binary sequence can be generated by the following:

(11) $$b(x_{i})=\left\{\begin{array}{cc} 0 & 0<x_{i}\leq 0.5\\ 1 & otherwise \end{array}\right.$$

### 5.1. Security Analysis of PRBG

#### 5.1.1. Key Space

To resist brute-force attacks, the key space should not be less than $2^{128}$. In the proposed PRBG, the parameter *u* and initial conditions $x_{0}$ and $y_{0}$ can be selected as secret keys. Given that 0<u<1, $0<x_{0}<1$, $0<y_{0}<1$, the key space is approximately equal to $10^{42}\approx 2^{140}$, which is enough to withstand all types of brute-force attacks.

#### 5.1.2. Key Sensitivity Analysis

Suppose that we only change the last digit of one of the keys and the other keys remain unchanged. Then, we observe its impact on the generated PRNG. We used the correlation coefficient and variance ratio to measure key sensitivity (as shown in Table 6 and Table 7). We made slight changes to the initial value of the system to generate two PRNGs. Table 6 shows that the correlation between the two PRNGs is very low, and Table 7 shows that their variance ratios are both close to 50%. Then, it turns out that, when the key is slightly changed, the generated sequence is very different from the previous one, which means that it has high key sensitivity when resisting brute-force attack.

#### 5.1.3. Linear Complexity

**Figure 21:**
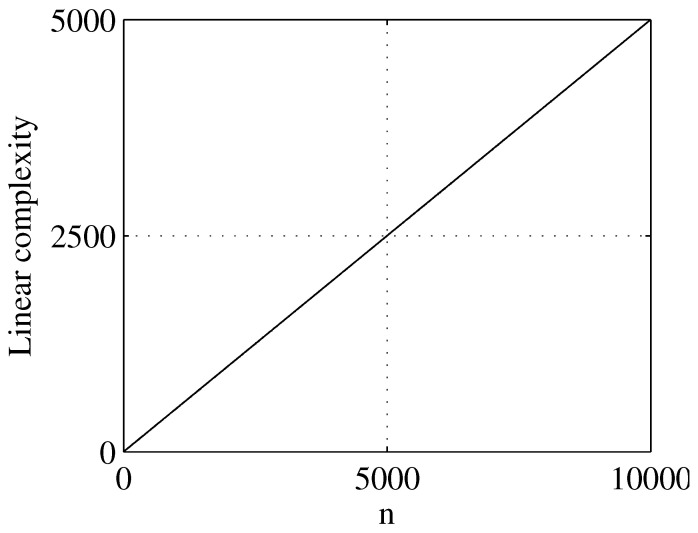
Linear complexity of the generated sequence.

Linear complexity is an important indicator to measure the randomness of pseudo-random sequences. The expectation of the linear complexity for a PRNG of length *n* is n/2. Figure 21 shows that the linear complexity of a binary sequence is approximately equal to the straight line n/2, which means that the generated sequence has a large linear complexity.

#### 5.1.4. Statistical Test

The NIST test suite is the most popular test suite for evaluating the randomness of pseudo-random sequences. These tests may be useful as a first step in determining whether a generator is suitable for a particular cryptographic application. Table 8 shows the NIST SP800 test results of the proposed PRNG. The test statistic is used to calculate a *p*-value that summarizes the strength of the evidence against the null hypothesis. For these tests, each *p*-value is the probability that a perfect random number generator produces a sequence less random than the sequence that was tested given the type of non-randomness assessed by the test. If a *p*-value for a test is determined to be equal to 1, then the sequence appears to have perfect randomness. A *p*-value of zero indicates that the sequence appears to be completely non-random. Table 8 shows that the proposed PRBG successfully passes all of the test indicators in the NIST test suite, which means the proposed PRBG has a high degree of randomness.

#### 5.1.5. Information Entropy Analysis

Table 9 presents the information entropy results with different initial values. Clearly, the information entropy is not affected by the change in initial value. Additionally, it stays close to 8, which means that the PRNG is almost impossible to leak information.

## 6. Conclusions

In this paper, we proposed a control method OCB for a digital Baker map based on feedback control technology. A continuous Chen chaotic system was introduced to control feedback to the digital Baker map to deal with its dynamic degradation. Three different control modes were investigated here, and it was concluded that the output control mode has a far better effect than the other two control modes in the case of low computing precisions. Then, an optimal control model was obtained by analyzing the influence of different gain coefficients on the properties of the controlled system. The optimal external feedback control can not only eliminate the dynamic bias in the system but also allows the digital system to display desirable dynamical properties, including ergodicity, a phase space with no pattern, impulse-like auto-correlation and close-to-zero cross-correlation, and ideal entropy values, etc. Performance comparisons with existing methods further showed the superiority of our method. Finally, we proposed a new PRNG with good randomness.

## Figures and Tables

**Table 1:** Approximate entropy of digital OCB systems with different gain coefficients.

*d*	−10	−0.001	0	0.0001	0.001	0.01	1	10
Approximate Entropy	2.1656	0.6936	0.7002	0.6043	0.6929	1.0552	2.1719	2.1615

**Table 2:** Information entropy of digital OCB systems with different gain coefficients.

*d*	−10	−0.001	0	0.0001	0.001	0.01	1	10
Information Entropy	7.9985	7.9961	7.2946	6.9404	7.9656	7.9980	7.9984	7.9980

**Table 3:** Permutation entropy of digital OCB systems with different gain coefficients.

*d*	−10	−0.001	0	0.0001	0.001	0.01	1	10
Permutation Entropy	0.9994	0.9962	0.7388	0.8571	0.9970	0.9994	0.9995	0.9994

**Table 4:** Information entropy values of improved systems for different methods.

System	Information Entropy
Our method	7.9980
DPM	7.3559
CCM	7.6917
DIM	0.0858
CPM	0.1114
BRM	4.2490
2D-SCS	4.5244
SJM	5.8792

**Table 5:** Permutation entropy values of improved systems for different methods.

System	Permutation Entropy
Our method	0.9994
DPM	0.9942
CCM	0.9994
DIM	0.0095
CPM	0.0123
BRM	0.4476
2D-SCS	0.4675
SJM	0.9278

**Table 6:** Correlation coefficients of the generated pseudo-random sequences with slightly different initial keys.

Initial Keys	Changed Keys	Correlation-Coefficient
$x_{0}$ = 0.2	$x_{0}$′ = 0.2 + $2^{-8}$	$C_{xy}$ = −9.8654 × 10${}^{-4}$
$y_{0}$ = 0.29	$y_{0}$′ = 0.29 + $2^{-8}$	$C_{xy}$ = 0.0011
$u_{0}$ = 0.49	$u_{0}$′ = 0.49 + $2^{-8}$	$C_{xy}$ = −0.1111 × 10${}^{-4}$

**Table 7:** Variance ratio when the key is changed by 1 bit.

Parameter	Variance Ratio
$x_{0}$	50.04%
$y_{0}$	49.93%
$u_{0}$	49.96%

**Table 8:** NIST SP800-22 test results of the proposed PRNG.

Test Index	*p*-Value	Results
Apen	0.5850	Success
Block-frequency	0.7221	Success
Cumulative-sums	0.5964	Success
FFT	0.8435	Success
Frequency	0.6194	Success
Linear-complexity	0.4982	Success
Longest-run	0.9853	Success
Nonperiodic-templates	0.7746	Success
Overlapping-templates	0.4532	Success
Random-excursion	0.1684	Success
Random-excursion-variant	0.5370	Success
rank	0.4137	Success
runs	0.6041	Success
serial	0.9018	Success
universal	0.1557	Success

**Table 9:** Information entropy values of the generated sequences with different initial values.

**Initial Values**	0.8147	0.0034	0.1270
**Information Entropy**	7.9998	7.9998	7.9998

## Data Availability

Not applicable.
